# Endocrine Disruptor Compounds in Environment: Focus on Women’s Reproductive Health and Endometriosis

**DOI:** 10.3390/ijms24065682

**Published:** 2023-03-16

**Authors:** Livia Interdonato, Rosalba Siracusa, Roberta Fusco, Salvatore Cuzzocrea, Rosanna Di Paola

**Affiliations:** 1 Department of Chemical, Biological, Pharmaceutical and Environmental Sciences, University of Messina, 98166 Messina, Italy; 2 Department of Veterinary Science, University of Messina, 98168 Messina, Italy

**Keywords:** endometriosis, endocrine disruptors, inflammatory issues, reproductive system

## Abstract

Endometriosis is an estrogen-dependent gynecologic illness that has long-term effects on a woman’s fertility, physical health, and overall quality of life. Growing evidence suggests that endocrine-disrupting chemicals (EDCs) may be etiologically involved in the development and severity of the disease. We consider the available human evidence on EDCs and endometriosis, limiting ourselves to studies that have individually assessed chemical amounts in women. Dioxins, BPA, Phthalates, and other endocrine disruptors, like DDT, are among the evidence indicating an environmental etiology for endometriosis. Collectively, this review describes how environmental toxins are linked to lower fertility in women, as well as a number of reproductive diseases, focusing on the pathology of endometriosis and its treatments. Importantly, this review can be used to investigate techniques for preventing the negative effects of EDC exposure.

## 1. Introduction

Endocrine disruptors are substances of various kinds, commonly named because they are considered capable of interacting in various ways with the endocrine system. To better understand their mechanism of action, we will explain the functioning of the endocrine system. The endocrine system is a collection of glands and cells that produce hormones and release them into the blood. Through blood circulation, hormones reach tissues and organs in every part of the body. The endocrine system controls growth, sexual development, sleep, hunger, and the way that the body uses food [1]. This includes, for example, the organs that release the sex hormones that regulate the female cycle, and the pancreas that produces insulin, the hormone that keeps blood glucose levels within limits [2]. Originally articulated in the early 1990s, the theory of endocrine disruptors proposes that some exogenous chemicals interfere with the endogenous hormonal axes [3]. In particular, these substances can interact in different ways in the human body and affect different districts. The nature of these substances is varied and their presence in the environment can be discovered and can include: different groups of pesticides, industrial substances such as bisphenol A and phthalates, and dioxins. The presence of EDCs in the environment, as well as their persistence in some other situations, has an influence on living things. Incorrect manufacturing and disposal methods are the primary contributors to environmental hazards, especially of industrial products containing glues, paints, and plastics [4]. The majority of exposures are caused by a wide range of chemicals and pollutants that are present in low amounts. The effects that endocrine disruptors have on humans can result from contamination through the food chain; as they accumulate mainly in soil and water, leading to the contamination of food and drink [5].

Being elements that can interact with the production of sex hormones, they also have an impact on the reproduction of different species, also interfering with biodiversity [6].

The use of environmental pollutants such as pesticides, and chemicals designed to repel or kill rodents, fungi, insects, and “weeds” that menace intensive farming, is a route to the exposure to ECDs [7]. Furthermore, pesticides play a role in a variety of chronic diseases, including neurological disorders, Parkinson’s disease, Alzheimer’s disease, diabetes, cancer, and reproductive issues [8,9,10]. Further side effects due to water contamination were analyzed in aquatic organisms; they include alterations in thyroid function resulting in impaired neuroendocrine development in the early stages of life, decreased survival of the offspring, and altered reproductive behavior in adulthood [11]. Furthermore, the phenomena of demasculinization and feminization, defeminization and masculinization, and sex inversion, which have been related to exposure to endocrine disruptors represent further signs of reduced fertility with possible consequent effects on the conservation of the species and the maintenance of the existing balance in ecosystems [12].

The mechanism of action of these substances is various: membrane receptors, the aryl hydrocarbon receptor, and enzymatic machinery involved in hormone metabolism are all possible targets for these compounds [13,14]. Moreover, their action is characterized by the interaction with cellular hormone receptors, preventing the action of endogenous hormones, acting as antagonists, or simulating the action of a natural hormone, excessively increasing the action. It is known that endocrine disruptors can act both with non-nuclear steroid membrane receptors and with non-steroid receptors, such as those found in the central nervous system, namely dopamine, serotonin, and noradrenaline [15]. However, the toxicity of some endocrine disruptors is given by the ability that these substances possess to interfere with hormonal signaling processes, mediated by nuclear receptors (NRs). The Aryl hydrocarbon/Dioxin receptor (AhR) has been defined as an environmental sensor and has also proven to be an important regulator of cell physiology and organ homeostasis. Recent studies have highlighted the action of AhR, in particular, the involvement of the receptor in the correct functioning of the immune, hepatic, cardiovascular, vascular, and reproductive systems. At the cellular level, AhR implements functional interactions with the signaling pathways that regulate the cell cycle and cell proliferation, adhesion, and migration [16]. Their diffusion is so pervasive as to make their exposure chronic and cause significant bioaccumulation in the food chain. The effects of ECDs on the human body are various and affect different systems; evidence also exists for relations between bisphenols and adult diabetes. Estrogenic receptors (ERα; estrogen receptors alpha) and ERβ (estrogen receptors beta) are the key parameters involved in glucose metabolism. So, when these receptors are attacked by natural or environmental chemicals, the effect is damage to the normal physiology of glucose homeostasis which can damage pancreatic cells, providing a clear indication of the onset of diabetes [8].

## 2. Endocrine Disruptors

### 2.1. Dioxin

**Figure 1:**
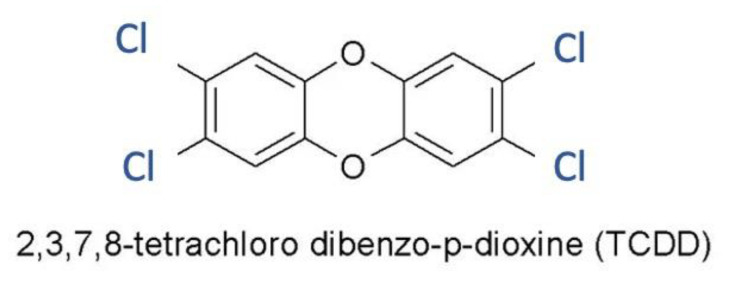
Chemical structure of TCDD.

The greatest exponent is represented by the 2,3,7,8-tetrachlorodibenzo-p-dioxin (TCDD) (Figure 1); dioxins are found in the environment as a result of garbage burning, particularly plastic trash. The fat-soluble goods most at risk of dioxin contamination include butter and fatty fish, milk, and its derivatives. Its prevalence in the environment is mostly owing to particle transmission in the air and accumulation in plants. Furthermore, because it is very resistant, it has the tendency to accumulate in soil and aquifers, polluting them. It will come to humans through food, mainly meat and dairy products. For this reason, the ingestion of contaminated food is the main source of exposure to the dioxin family [17]. 

In the family of dioxins and dioxin-like, the most hazardous substance is TCDD, which works by interacting with the steroid receptor, it also interferes with the metabolism and transport of the steroids themselves. TCDD reduces the uptake of glucose in adipose tissue and reduces insulin secretion. Moreover, it can induce polycystic ovarian syndrome, and reduce semen quality; phthalates, for example, prematurity, and obesity [18]. The activity of this molecule has been evaluated on the female reproductive system and it has been demonstrated that, in immature female rats, it alters the concentration of estradiol, FSH, and LH [19].

The strong affinity connection that dioxin has with the aryl hydrocarbon/dioxin receptor (AhR) determines the harmful consequences at the cellular level which, when followed, will give rise to a heterodimeric complex activated with the nuclear transport protein of the aryl hydrocarbon structurally related (ARNT) [20]. 

### 2.2. BPA

**Figure 2:**
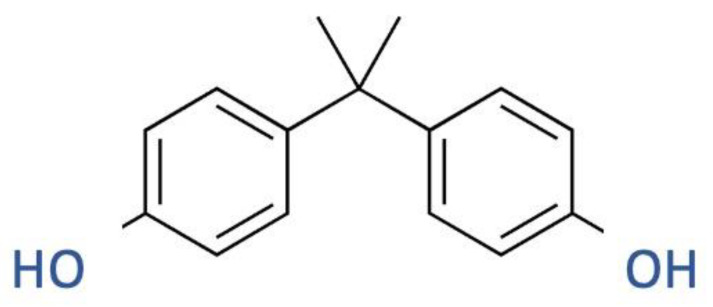
Chemical structure of BPA.

Bisphenol A (BPA) (Figure 2) is a xeno-estrogen that is currently utilized as a basic ingredient in the manufacture of polycarbonate plastic and the resin lining of food and beverage cans and drinking water bottles. The toxicity of BPA can be related to its lipophilicity, as it gives the molecule the ability to accumulate in the tissues; in fact, analysis of human serum concentrations found different levels of toxicity [21]. It can cross the placenta, and, in some vivo experiments, has shown that it can cause unfavorable neonatal outcomes in offspring. For example, oral administration of 10 mg/kg per day of BPA to pregnant rats may cause a decrease in the number of births and the neonatal survival rate [22]. In-utero exposure to phthalates and BPA was associated with delayed pubertal development in females, especially if the normal weight, and earlier pubertal development in males, especially if overweight/obese [22]. Higher urine concentrations have been linked to an increased risk of cardiovascular disease, type 2 diabetes, abnormal liver enzymes, and obesity in people. High concentrations of BPA may be related to abnormalities in the concentrations of some liver enzymes, such as γ-glutamyltransferase and alkaline phosphatase. BPA, administered orally, is rapidly metabolized and excreted; in fact, its metabolite BPA-monoglucuronide is easily found in the urine, a source of evaluation for exposure to this type of interferent. BPA is soluble in water and this can lead to accumulation phenomena in the intestinal wall and in the liver, to be then rapidly excreted by the kidneys via the urine. BPA has a short half-life, approximately 6 h, following oral administration [23].

BPA is an environmental and food contaminant that can affect lipid and glucose homeostasis in various tissues by interfering with different nuclear receptors involved in the regulation of metabolism; in fact, it is defined as an obesogenic agent [24]. Peroxisome Proliferator-Activated Receptors PPARs (a, b/d, and c isoforms) are a family of ligand-inducible transcription factors that play key roles in modulating the expression of genes involved in lipid homeostasis. The levels of BPA in the adipocytes were studied, in which the effects found at high doses included an increase in differentiation, an increase in genes involved in lipid metabolism, and stimulation of the accumulation of the same. Adipocytes are the main cellular component of adipose tissue. Adipocytes from the 3T3-L1 line have been frequently utilized to study adipocyte physiology [25].

### 2.3. Phthalates

Phthalates are rapidly metabolized and excreted in urine and feces; the generally polar and low molecular weight phthalates metabolize into their hydrolytic forms by hydrolysis of one of the ester linkages during the initial phase of biotransformation. Instead, the metabolism of high molecular weight phthalates first involves conversion to hydrolytic monoesters and, subsequently, to hydrophilic molecules, after enzymatic oxidation of the alkyl chain (Table 1).

Phthalates are used as plasticizers in PVC plastics. At high doses, they produce antiandrogenic effects by reducing testosterone production and perhaps reducing estrogen production. Di-(2-ethylhexyl) phthalate (DEHP) is one of the most widespread phthalate plasticizers; some phthalates are reproductive and developmental toxicants in animals and suspected endocrine disruptors in humans. The toxic effects are mediated by the induction of the PPAR-alpha receptor [26].

Recent studies, conducted on experimental animals, have highlighted the correlation between exposure to DEHP and its metabolite ormono-ethylhexyl phthalate (MEHP) and, mainly in mice, ovarian toxicity in sexually active subjects. DEPH exposure reduced estradiol concentration levels in follicle granulosa cells in the preovulatory phase in a study conducted 8 days after exposure. The reduction in serum levels of estradiol produced an increase in the concentration of the hormone FSH, reducing those of the hormone LH essential for ovulation. As a result, ovulation did not occur in the DEHP-treated rats [27]. Another study showed that women with endometriosis had higher plasma levels of DEHP, and 92.6% had a detectable level of DEHP and MEHP in the peritoneal fluid; phthalate metabolites were found to be higher in the urine of women who subsequently delivered [28]. Thomsen et al. reported that women exposed to MEP showed adverse effects of some of the phthalates and their metabolites on female reproduction and pregnancy outcomes [29].

**Table 1:** Example of phthalates.

Compounds	Sources	Exposition Effects
di-(2-ethylhexyl) phthalate (DEHP)	Perfumes, PVC plastics used in household products (e.g., toys, floor tiles and furniture upholstery, cables, garden hoses, wall coverings, and gloves), food packaging, blood storage bags, and medical devices.	[30]
diethyl phthalate (DEP)	Used as solvents and fixatives in fragrances, additives in cosmetics, medical devices, and household and personal care products.	[31]
di-*n*-butyl phthalate (DBP)	Cellulose acetate plastics, solvent for oil-soluble dyes, pesticides, personal care products (e.g., nail polish and cosmetics), lacquers, varnishes, and coatings (e.g., pharmaceuticals).	[32]

### 2.4. Pesticides 

Among the endocrine disruptors present in the environment, we find pesticides. The current data shows that about 2.5 million tons of pesticides enter the environment each year worldwide [33]. Pesticides are toxic chemical substances that are intentionally released into the environment to control pests and weeds; it is a technique used in both developed and developing countries. [34]. Pesticides are critical in modern agriculture since, without them, up to 50% of crops in tropical warm climate regions could perish [35]. The widespread use of pesticides has caused serious aquatic pollution that can be dangerous for human health [36]. Pesticides have a high propensity to accumulate in the environment, have lipophilic qualities, and have a lengthy half-life and long-term transfer ability, which has led to extensive research into their environmental consequences [37]. Pesticides can enter the human body through processes of inhalation or penetration through the skin, but the highest toxicity is given by the ingestion of contaminated food or water [38]. For humans, sources of pesticides may be fish, meat, and dairy products (containing high lipid fractions), as well as drinking water, indoor and ambient air, dust, and soil [39]. Toxic effects are produced when the concentration of pesticide in the body increases far more than its initial concentration in the environment; to confirm human exposure to pesticides, analyses are performed on biological samples such as serum/plasma, milk, and adipose tissue [40]. Among the best-known pesticides, we find Dichlorodiphenyltrichloroethane (DDT); for its nature, the metabolite of the DDT associates with lipids and accumulates in adipose tissue [41]. The long-term presence of pesticides in the human body has led to an alteration of reproductive capabilities, altering the levels of male and female reproductive hormones; consequently, this results in stillbirth, birth defects, spontaneous abortion, and infertility [38]. An example of a pesticide that has an action on the reproductive system is DDT; in fact, this agent has antiandrogenic and estrogen-like properties [42]. A study conducted on rats showed that daily exposure to DDT in prenatal and postnatal development periods produced a variation in male and female sex steroids probably determined by both the direct disruptor action of DDT and the action of the hypothalamic-pituitary system [42]. Another study showed that the disruptor effect of DDT is not only associated with the competition with testosterone to bind to androgen receptors and impair receptor signaling in target cells, but it also implicates an increase in estrogen synthesis [43]. The revealed alterations in sex hormone production suggest reproductive and somatic disorders in later life since the hyperproduction of estrogens and an unbalanced testosterone/estradiol ratio are associated with an increased risk of feminization and are also known to trigger metabolic disorders as well as estrogen-related cancers and cardiovascular diseases [44]. 

## 3. Epidemiological Studies

Evidence from epidemiologic research has been used to support numerous EDC-specific health-based choices, including the prohibition of BPA use in baby bottles [45] (Table 2). Close cooperation across scientific disciplines is required to enable the creation of such preventive measures and the assessment of their health impact and epidemiologic data is particularly included among risk assessment methodologies in certain jurisdictions to investigate EDCs [46]. 

## 4. Endocrine Disruptors and Women’s Reproductive System

The reproductive system is the primary target of most endocrine disruptors [59]. The ubiquity of ECDs has led to the onset of forms of cancer in women; in particular, cases of cancers of the reproductive organs, such as breast and ovarian, which seem to increase [60]. In particular, it is probable that intrauterine influences can influence the initial processes leading to the growth of tumors many years later. There are “critical windows” in fetal and newborn development when the body is particularly susceptible to the estrogen/androgen ratio and steroid levels, according to epidemiological and nutritional research [61]. There are factors that influence ovarian formation, which interfere with the migration of germ cells into the yolk sac in the first trimester, with differentiation into oocytes in the second and third quarters. This can lead to an impact on reproductive outcomes decades later, just like any other postnatal dysregulation of uterine formation [62]. Studies showed that urinary concentrations of the metabolite monoethyl phthalate can produce infertility and urinary levels of this metabolite were significantly associated with clinical pregnancy loss and preterm labor [63]. 

DEHP is the most often used plasticizer in PVC; DEHP induces embryotoxic and teratogenic effects in mice and rats when exposed in gestation [64]. Furthermore, a study in monkeys found a dose-dependent relationship between TCDD and dioxin-like polychlorinated biphenyls (PCBs); in particular, the study was conducted in a chronic rhesus monkey colony exposed to TCDD or dioxin for a period of 4 years. The presence of endometriosis was documented by laparoscopy 10 years after dioxin treatment was stopped, and the severity of the disease was examined. Endometriosis was found to be directly linked to dioxin exposure, and the severity of the condition was determined by the dose given [65]. Some adverse reactions due to exposure to DEPH were highlighted; because lowering of estradiol levels is the principal functional alteration of DEHP in adult female rats, structurally similar phthalates were examined for their effects on estradiol. In vivo, the research looked at serum estradiol and estrone, the principal metabolite of estradiol, to see if metabolism was linked to lower serum levels of estradiol as well as lower estradiol synthesis [64]. The pituitary hormones FSH and LH, which stimulate granulosa and thecal cells, respectively, govern ovarian hormone production in vivo, Monoethylhexylphthalate reduced FSH-stimulated cAMP and progesterone synthesis in rat ovarian granulosa cell cultures [66].

## 5. Endocrine Disruptors and Endometriosis

Endometriosis is a gynecological disease, characterized by a chronic inflammatory condition in which there is the presence of endometrial tissue outside the uterus. The prevalence of endometriosis is difficult to determine accurately; laparoscopy or surgery is required for the definitive diagnosis. The clinical symptoms of endometriosis include dysmenorrhea, pelvic pain, dyspareunia, infertility, and pelvic mass [67]. It is estimated that endometriosis affects approximately 176–200 million women worldwide, more than 10% of women of reproductive age, which can reach up to 40% of women aged 18–44 years and undergoing pelvic surgery [20,68].

There is evidence that lifestyle exposures that may elevate or lower estrogen levels may alter risk, such as a lower risk associated with smoking and exercise and a higher risk related to caffeine or alcoholic beverages. These risk factors appear to be consistent with the central importance of retrograde menstruation, which can be affected by outflow blockage, immunological factors, or hormonal stimuli [69].

Often, infertile patients have no painful symptoms, and their disease is only discovered in the course of the diagnostic work-up for infertility. The reason for these differences in clinical manifestations is still unknown [70].

Endometriosis is associated with a significant economic burden in addition to its impact on women’s health. Although the exact etiology of endometriosis is unknown, the most common theory suggests that retrograde menstruation is to blame. The data that endometrial tissue injection or seeding into the peritoneal cavity of rodents and baboons can cause disease development supports this idea. This tissue must first adhere, penetrate the mesothelium, develop a vascular supply, and multiply after being injected into the peritoneal cavity [71]. This condition causes pelvic pain and infertility in around 5% of women; several pieces of evidence point to a hereditary aetiology for this disease, especially with 51% heritability [72]. The uterus is a muscle organ consisting of two main elements: the body, which includes the endometrium, and the caudal end, or cervix, both exposed to significant hormonal influence from their early stages of development. The uterus undergoes periodic hormonal changes after puberty and dramatically grows during pregnancy, whereas the uterus involutes and shrinks after menopause. The endometrium is one of the targets of endocrine disruptors; the endometrium undergoes cyclical patterns of endocrine and immune signaling, essential for regulating growth and function during the reproductive years. As a result, it is particularly susceptible to substances that could alter the endocrine system.

Endometriosis is well known to be impacted by steroid hormones. Endometriosis lesions do, in fact, have estrogen and androgen receptors, and estrogens can help them proliferate. The impact of environmental compounds having estrogenic activity, often known as endocrine disruptors, on the female reproductive system throughout development is becoming increasingly popular [73]. Several gynecologic disorders have been linked to exposure to various environmental toxins, particularly when exposure occurs during critical stages of development. BPA is the first synthetic chemical to cause selective estrogen receptor modification, in particular, pollutants with hormone-like action. It was recently discovered that prenatal BPA exposure can cause an endometriosis-like phenotype in mice [74]. Phthalates and Bisphenol A (BPA) are chemicals present in a number of products, including food packaging and home items. Phthalates and BPA are examples of endocrine disruptors, which can be found in a variety of substances and are regularly exposed to the general public. Inhalation, ingestion, and cutaneous contact are all possible routes of exposure [75]. Urine analysis is a viable approach for determining human exposure to these chemicals; in fact, endometriosis is one of the disorders considered to be linked to high levels of phthalate metabolites and BPA metabolites in biological fluids [76]. The implantation and development of endometrial cells present in retrograde menstruation cause ectopic lesions. The status of endometrial debris is linked to the progression of lesions. We predicted that BPA exposure leads to endometriosis development by upregulating ERβ (estrogen receptors beta) expression in eutopic endometrium via an H3K4me3-related epigenetic mechanism, based on the background reported above [77]. 

TCDD is the most dangerous of the environmental toxicants in the dioxin and dioxin-like family [78,79]. Its mechanism of action has been seen to involve interference with the metabolism and transport of steroids, as well as a reduction in the expression of steroid receptors. The effects that we can see at the cellular level concern the high-affinity binding of TCDD with the AhR receptor which subsequently forms a heterodimeric complex activated with the structurally related protein ARNT. As can be seen in the adipose tissue near the genital tract of type Balb-c-adult female rats, exposure to BPA in the gestational and neonatal periods causes the development of endometrial glands and stroma [80]. Numerous studies have been conducted to evaluate toxic effects when exposure occurs in the neonatal period in rodents in order to explore the role of early life exposures on the adult development of endometriosis phenotypes. One of the earliest studies examining the potential role of developmental toxicant exposure and endometriosis was conducted by the Birnbaum laboratory. Birnbaum and colleagues conducted an experiment in which they exposed both mice and pregnant rats at day 8 of embryonic (E8) to TCDD and then surgically induced endometriosis in adult offspring followed by a second. This research backs up their previous findings, which showed that murine models of experimental endometriosis are more responsive to TCDD than rats using acute exposures [81]. Currently, endometriosis is the subject of many studies, as it is considered a chronic inflammatory disease characterized by the presence of enhanced numbers of activated, peritoneal immune cells but also by the negative impact of activated immune cells on overall reproductive success. Endometriosis is thought to be the result of an immune cell response to an infection combined with the oxidative stress associated with menstruation, resulting in a chronic, sterile inflammation that promotes illness. Early life exposure to an EDC such as TCDD may simulate an infection, resulting in persistent inflammation and an elevated risk of disease development, according to research [20]. Some studies have been conducted in vitro to evaluate the toxicity of endocrine disruptors on cell cultures. They have been implicated in reproductive disturbances; in particular, the action that the AhR receptor was focused upon. Physiologically, AhR is found in the cytosol in association with heat shock protein (HSP) 90 and HSP90 accessory proteins; when the ligand binds to the receptor, it is freed from the complex and promotes nuclear translocation. In the nucleus, the ligand-activated AhR forms a heterodimeric complex with the aryl hydrocarbon nuclear translocator (ARNT). Among the contaminants, the most persistent in the environment is an AhR agonist, TCDD; we find it in the environment because it is the product that can be obtained from the incineration of waste containing chlorine, from the production of some pesticides, and the bleaching of paper pulp. TCDD has anti-estrogenic effects in the rat uterus and in the MCF-7 breast cancer cell line [82]. Human endometrial endothelial cells (HEECs) express estrogen receptor beta (ER) and progesterone receptor (PR). Critchley demonstrated immunoreactive ER-β, but not ER-α, in the nuclei of endothelial cells from most spiral arteries, capillaries, and veins of the human and nonhuman primate endometrium. Steroid receptor expression may potentially make HEECs vulnerable to endocrine disruptors [83]. The transcription factor nuclear factor-kappa B (NF-kB), is activated during inflammatory processes; in women suffering from endometriosis, this factor is activated and, to ascertain the inflammatory state typical of the disease, at the level of endometriostic implants, the expression of the intercellular adhesion molecule (ICAM) -1 was studied in the peritoneal endometriotic lesions according to their type. In addition, the p65 and p50 subunits of active NF-kB dimers were examined in endometriotic lesions in order to obtain insight into NF-kB-implicated pathways [70]. In this study, women with peritoneal endometriosis had constitutive NF-kB activation. The involvement of p50/p65 dimers in NF-kB activation reveals a role in the conventional NF-kB activation pathway in endometriosis, making it a promising therapeutic target [84].

## 6. Endometriosis and Treatment

Endometriosis is a condition in which functional endometrial glands and stroma are present outside the uterine cavity, which can be an enigmatic and frustrating condition for both patients and physicians. While this condition has been recognized since the late seventeenth century, pathogenesis has not been completely clarified. The diagnosis of endometriosis may be difficult, and therefore the treatment of this condition requires individualized planning, following a complete evaluation (Table 3). The main goals of treatment are to remove the majority of endometriotic implants, restore normal anatomy, prevent or postpone progression, and alleviate symptoms. Assisted reproductive procedures have been used to treat infertility caused by endometriosis. These include the induction of ovulation with clomiphene or gonadotropins, the induction of ovulation combined with intrauterine insemination, and more advanced techniques such as in vitro fertilization, gamete intrafallopian transfer, and zygote intrafallopian transfer [85]. Oral contraceptives with high and low doses have been used for the induction of pseudo-pregnancy states. This therapy can be useful for women whose main symptom is pain. A study compared a low-dose contraceptive with a GnRH agonist and was conducted on 57 women. The women participating in the study had moderate to severe pelvic pain associated with laparoscopically confirmed endometriosis. Women receiving the contraceptive experienced a significant reduction in dysmenorrhea and nonmenstrual pain [86]. Another example consists of a glucocortical agonist. It produces an amenorrheic state with higher than normal serum androgen and low serum estrogen concentrations. Due to this androgenicity, a number of side effects can occur, and its use, therefore, has declined lately [86]. Since the majority of endocrine disruptors (EDC) exposure occurs via diet, choosing organic foods, lean meats, or a vegetarian lifestyle can help to minimize exposure. Additionally, reducing the usage of canned foods containing a BPA liner, using BPA/BPS-free products, and avoiding either long-term storage or heating of foods in plastic containers will also reduce incidental EDC exposure. Anti-inflammatory agents are an attractive therapeutic option to explore. Furthermore, since many women with endometriosis wish to preserve their fertility or to actively pursue pregnancy during treatment, dietary agents with anti-inflammatory properties are being explored for their utility in treating this disease. Resveratrol, for example, is a polyphenolic compound and natural phytoestrogen with anti-inflammatory effects and antiproliferative activity. Mechanisms of action of resveratrol further include multiple cellular targets affecting various signal transduction pathways, including AKT, RPS6KB2 (p70S6K), mitrogen-activated protein kinase 1/3 (MAPK1/3; ERK1/2), STAT3, MAPK14 (p38), protein kinase C, and peroxisome proliferator-activated receptors (PPAR) gamma. Importantly, several of these pathways are relevant to the pathophysiology of endometriosis, especially in relation to their impact on inflammatory processes [87].

## 7. Conclusions

In this review, the focus was on endometriosis, a chronic inflammatory condition in which there is the presence of endometrial tissue outside the uterus. The clinical symptoms of endometriosis include dysmenorrhea, pelvic pain, dyspareunia, infertility, and pelvic mass. The diagnosis of the disorder can only be made by laparoscopy. In particular, our attention was focused on the ability of endocrine disruptors to induce the disease, with prolonged exposure over time. Endocrine disruptors are a group of substances of various kinds, so defined because they are able to interact with the human organism, in particular the endocrine system. Their presence in the environment feeds contamination and, consequently, the possible onset of endometriosis. We can find endocrine disruptors in the air, in water, but also in plastics and products for preserving food; in fact, it is their ubiquity that makes exposure to these substances easier. In humans, they can arrive, as well as from the air, through exhaust fumes, cigarette smoke, and also through food, due to contamination of aquifers and food. The mechanism of action of these substances is various and includes interaction with membrane receptors, the aryl hydrocarbon receptor, or the enzymatic machineries involved in hormone metabolism. Studies conducted both in vitro and in vivo have shown that some endocrine disruptors, including TCDD, are capable of inducing the disease. To date, the most effective treatment is surgery, in which most or all of the endometriotic implants are removed in order to restore normal anatomy, prevent or delay progression, and relieve symptoms. Drug therapies include treatment with contraceptives and glucocorticoid agonists, but new substances have been targeted for their mechanism of action which leads to the reduction of inflammation and antiproliferative activity. Future perspectives in the treatment of endometriosis may include studies on molecules capable of exerting an anti-inflammatory and antiproliferative action.

## Figures and Tables

**Table 2:** Example of descriptive, causal, predictive, and evaluative questions in epidemiologic studies on EDCs.

Example of Research Question	Example of Study Objective	Study Examples
What environmental contaminants are pregnant women exposed to during pregnancy?	To characterize concentrations of a large number of environmental contaminants in pregnant European women.	[47,48,49,50,51,52,53]
What proportion of male reproductive disorders and diseases would be prevented by a ban on EDCs in the European Union?	To estimate the incidence and prevalence of selected male reproductive disorders and diseases attributed to EDC exposure in the European Union.	[54,55,56]
Can using “low-chemical” personal care products significantly lower levels of EDCs in the body?	To evaluate whether changing personal care products to those labeled as “low chemical” reduces urinary concentrations of metabolites of phthalates, parabens, and phenols in adolescent girls.	[57,58]

**Table 3:** Endometriosis treatment.

Example of Research Question	Example of Study Objective	Study Examples
Are Opioids a Possible/Promising Treatment for Endometriosis?	The mechanisms of pain development in endometriosis disease, the endogenous opioid system and pain, as well as the opioid receptors and endometriosis-associated pain.	[88]
Does the surgical treatment of endometriosis in conjunction with assisted reproduction improve pregnancy rates?	The year following the assisted reproductive technology (ART) cycle, a significantly higher spontaneous pregnancy rate was seen in those undergoing surgery.	[85]
Robotic surgery or laparoscopic surgery?	The study aimed to evaluate the safety and efficacy of robotic-assisted laparoscopic surgery (RAS) versus conventional laparoscopic surgery (LPS) in the treatment of endometriosis. This meta-analysis demonstrated that robotic surgery is safe and practical in endometriosis patients. We may argue that RAS is a viable option that could be regarded as an alternative to LPS, particularly in advanced situations.	[89]

## Data Availability

Not applicable.
